# Circulating Endothelial Cells in Refractory Pulmonary Hypertension in Children: Markers of Treatment Efficacy and Clinical Worsening

**DOI:** 10.1371/journal.pone.0065114

**Published:** 2013-06-10

**Authors:** Marilyne Levy, Damien Bonnet, Laetitia Mauge, David S. Celermajer, Pascale Gaussem, David M. Smadja

**Affiliations:** 1 Université Paris Descartes, Sorbonne Paris Cité, Paris, France; 2 AP-HP, Hôpital Necker-Enfants Malades, Centre de Référence Malformations Cardiaques Congénitales Complexes (M3C), Paris, France; 3 Inserm UMR-S765, Faculté de Pharmacie, Paris, France; 4 AP-HP, Hôpital Européen Georges Pompidou, Service d’hématologie Biologique, Paris, France; 5 Sydney Medical Schools, University of Sydney, Sydney, Australia; Vanderbilt University Medical Center, United States of America

## Abstract

**Background:**

Pulmonary vasodilators in general and prostacyclin analogues in particular have improved the outcome of patients with pulmonary arterial hypertension (PAH). Endothelial dysfunction is a key feature of PAH and we previously described that circulating endothelial cell (CEC) level could be used as a biomarker of endothelial dysfunction in PAH. We now hypothesized that an efficient PAH-specific vasodilator therapy might decrease CEC level.

**Methods/Results:**

CECs were prospectively quantified by immunomagnetic separation with mAb CD146-coated beads in peripheral blood from children with idiopathic PAH (iPAH, n = 30) or PAH secondary to congenital heart disease (PAH-CHD, n = 30): before, after treatment and during follow up. Controls were 23 children with reversible PAH. Oral treatment with endothelin receptor antagonists (ERA) and/or phosphodiesterase 5 inhibitors (PDE5) significantly reduced CEC counts in children. In 10 children with refractory PAH despite oral combination therapy, subcutaneous (SC) treprostinil was added and we observed a significant decrease in CEC counts during the first month of such treatment. CECs were quantified during a 6 to 36 month-follow-up after initiation of SC treprostinil and we found that CEC counts changed over time, with rising counts always preceding clinical deterioration.

**Conclusion:**

CECs might be useful as a biomarker during follow-up of pediatric iPAH and PAH-CHD to assess response to treatment and to anticipate clinical worsening.

## Introduction

Pulmonary arterial hypertension (PAH) is due to progressive pulmonary vascular disease and may eventually lead to right heart failure and death [1]. In recent years, the available data on targeted PAH therapies suggest that they are generally well tolerated and effective in children, which is generally the case in adults [2], [3]. In adult patients, several studies have addressed the question of combined therapy at the time of clinical worsening [4]. These studies consist mostly of add-on therapy and have suggested additional beneficial effects [5]. Still, the optimal timing of combination therapy remains unclear. Hoeper et al. developed a treatment algorithm based on the concept of “goal-oriented” treatment and showed that this led to improved outcomes in their patients [6]. Whether or not this approach is applicable to children with PAH remains uncertain. Furthermore, dose formulation and the role of prostacyclin analogues are also challenging issues in the management of children with PAH. Decision-making often varies between centers [2], [3], [7] and NYHA functional evaluation is also difficult in younger children. For these reasons, biomarkers of treatment response and/or predictors anticipating any change in clinical status would be of great value in managing children with PAH.

Recently, we have shown that counting circulating endothelial cells (CECs) could be helpful in identifying children who may benefit from closure of a left-to-right shunt responsible for PAH, compared to children with irreversible PAH [8]. CEC counts have been shown elevated in adult idiopathic PAH as well [9], [10]. In a prospective study, we therefore aimed at determining if CEC count might change with PAH targeted therapies in children. Our second aim was to count CECs in peripheral blood during follow-up, to assess the relationship between CEC counts and change in clinical status.

## Patients and Methods

### Study Population

The Institutional Ethics Committee approved this study and signed informed consent was obtained from parents in all cases. Thirty consecutive patients with Congenital Heart Disease (CHD) and elevated pulmonary vascular resistance (irreversible PAH) and thirty consecutive patients with idiopathic PAH (iPAH) were enrolled at the Necker-Enfants Malades Hospital between February 2008 and December 2010. All clinical decisions were made by the attending physician as a part of routine care, independently of the research study.

Twenty-three children with reversible PAH-CHD (patients that normalized their pulmonary pressure after shunt closure) were used as a control population as we have previously shown that CEC level in this population is normal, compared with healthy age matched controls [8].

Table 1 shows the patients’ clinical characteristics. All patients had a right heart catheterization (RHC) and complete pulmonary hypertension workup including functional assessment, 6-min walk test (6MWT) (when appropriate), Brain natriuetic petide (BNP) and echocardiography. In the 30 patients referred to our institution with idiopathic PAH, 15 of them were explored without any treatment while 18/30 patients with irreversible PAH where explored. Thus, from the 60 patients, 33 were explored before any PAH treatment and 27 had previously started PAH treatment. Patients with Down syndrome were not included.

**Table 1:** Characteristics of the patients.

	Controls (Reversible PAH, n = 23)	Irreversible PAH (n = 30)	Idiopathic PAH (n = 30)
**Age (y)**	2 (1–26)	6 (1–53)	7.5 (1–21)
** Difference vs reversible PAH**		0.02*	0,19
** Difference vs irreversible PAH**			0,36
**Saturation (%)**	96 (75–100)	92 (66–100)	97 (79–100)
** Difference vs reversible PAH**		0,1	0,52
** Difference vs irreversible PAH**			0.04*
**Mean PAP**	55 (20–70)	65 (25–98)	53 (27–88)
** Difference vs reversible PAH**		0.01*	0,34
** Difference vs irreversible PAH**			0,14

Data are expressed as medians and their range. Baseline characteristics were compared between the groups by using Wilcoxon’s rank sum test for none normally distributed variables (age) and Student’s unpaired test otherwise. Symbols: * p<5%.

### Therapeutic Strategy

Our usual strategy to treat children with PAH consists of treating all children in Functional Class (FC) II or higher [2], starting firstly with oral monotherapy. In the present study, 52 patients received oral monotherapy (bosentan n = 24 or sildenafil n = 26 according to individual physician preference and calcium channel blockers in 2 NO responder patients). In cases of worsening or non-improvement, a second oral drug was added and, finally, prostacyclin analogues on top of oral bi-therapy were given to 10 patients.

Clinical worsening was prospectively defined as syncope or the combination of two of the following items:

global clinical impression of physician in charge or parents
20% decrease in the 6MWT
Deterioration of right ventricular function on echocardiography
Increase in brain natriuretic peptide (BNP)
New pericardial effusion.

At the time of worsening, we added a second oral drug, after a repeated right heart catheterization (RHC) to confirm either an increase in PVR or a decrease in cardiac output. We also added a second oral drug in case of "no improvement" but, in these circumstances, RHC was not systematically repeated, in such patients of our series.

In cases of no improvement or worsening with combined oral therapy, we added subcutaneous treprostinil (which is the first choice in our institution when prostanoïds are needed), as previously reported [7]. In addition to these patients who received sequential tritherapy, two children in FC IV received combined tritherapy (sildenafil-bosentan-treprostinil) up-front. The characteristics of the 10 patients (median age 5.5 years, range 1.2–13 years) who received tritherapy are given in table 2.

**Table 2:** Characteristics of the patients treated with SC-treprostinil.

Patient	Sex	Cause of PAH	Age at diagnosis of PAH	Age at initiation of first treatment	Age at initiation of dual therapy	Age at initiation of SC treprostinil over both oral therapy	Oxygen saturation	NYHA functionnal class
1	M	Postoperative, TGA	3 months	7 months, bosentan	19 months, sildenafil	4 years	95%	III
2	M	Postoperative PDA	3.5 years	3.5 years, sildenafil	4 years, bosentan	5.5 years	86%	III
3	F	Small defect, VSD	2 months	9 months, sildenafil	10 months, bosentan	2.5 years	98%	III
4†	F	Small defect, ASD	6 years	7 years, bosentan	7 years, sildenafil	10 years	71%	IV
5	M	iPAH (Heritable)	4 months	5 months, sildenafil	6 months, bosentan	15 months	95%	III
6†	M	iPAH	3 months	4.5 years, bosentan	6.5 years sildenafil	10 years	89%	IV
7	F	iPAH	2 years	2 years, IV epoprostenol	5 years bosentan 7 years, sildenafil	9 years	98%	II
8	M	iPAH	16 months	16 months, bosentan	22 month, sildenafil	2.5 years	88%	IV
9	F	iPAH	12 years	12 years, directly tritherapy	/	12 years	94%	IV
10	F	iPAH	4 years	4 years	4 years sildenafil and bosentan	5 years	98%	IV

*TGA*, transposition of the great arteries; *PDA*, patent ductus arteriosus; *VSD*, ventricular septal defect; *ASD*, atrial septal defect.

† Deceased.

### Follow-up Methods

All patients were evaluated one month after treatment initiation or modification and every six months when in stable condition, after they had improved their clinical status for a period lasting at least 3 months. Stable condition was defined as FC I or II, increased 6MWT of more than 20% compared to baseline, no worsening of right ventricular dysfunction on echocardiography, low level of BNP no pericardial effusion and normal cardiac output on RHC. Systematic RHC was performed in all children within the first year after treatment initiation. RHC was also routinely performed when patients worsened before any treatment change was undertaken. All patients but the three youngest underwent the 6-minute walk test (6MWT) before initiation of treprostinil treatment. In all patients, BNP levels were measured before and after treatment. Values are given in Table 3. The three patients who died had significantly higher BNP levels compared with the other patients. Patient follow-up has been reached 3 years for treprostinil treated patients.

**Table 3:** Effect of SC treprostinil therapy on functional and hemodynamic status.

Patient N°	1		2		3		4†		5		6†		7		8		9		10	
Follow-up (months)	24		18		20		2		21		6		36		6		12		12	
	before	after	before	after	before	after	before	after	before	after	before	after	before	after	before	after	before	after	before	after
SaO_2_	92	100	86	97	90	98	70	75	100	100	98	98	99	99	88	97	94	98	98	98
NYHA	III	I	III	II	III	II	IV	IV	III	II	IV	III	II	II	IV	II	IV	I	IV	II
6MWT(m)	110	360	140	360	200	420	240	250	–	–	–	–	400	520	–	–	100	570	200	470
BNP pg/mL	27	35	8	5	39	14	1220	837	47	8	283	1150	28	28	480	400	382	5	6	5
PAP mean(mmHg)	63	71	60	63	40	60	60	–	83	103	65	–	60	55	60	60	68	68	72	70
SAP mean(mmHg)	73	83	56	64	63	63	58	–	85	80	60	–	75	91	55	55	85	87	85	70
CO(L/min)	2,6	3,6	4,7	4,8	2,6	2,3	3,7	–	2,2	3,3	2,2	–	3,6	4	–	–	2,3	5,2	3	2,1
PVR(Woods Units)	20,4	15,8	11,5	11	21,4	23	19	–	35,5	28,8	24,8	–	14,8	11,5	19,9	–	25	12	21	7

† Deceased.

### CEC Counting after Immunomagnetic Separation

Peripheral venous blood samples were collected on EDTA after having always discarded the first milliliter of blood to avoid presence of endothelial cells dislodged by puncture. CECs were isolated by immunomagnetic separation with mAb CD146-coated beads and staining with the fluorescent probe acridin orange or Ulex-Europaeus-Lectin-1, as previously described [8], [10], [11], [12]. An operator unaware of patient’s clinical features counted CECs.

CEC levels were performed at basal evaluation and during follow-up. When treated, the patients were evaluated one month after treatment initiation or modification and every six months thereafter.

### Statistical Analysis

Subjects’ baseline characteristics were analyzed by using Wilcoxon’s rank sum test for non- normally distributed variables and Student’s unpaired t-tests otherwise. Group effect and their interaction with CEC variability was tested by using two-way repeated measure analysis of variance (ANOVA) on CEC numbers. Comparisons between groups were confirmed using independent samples Student *t* tests. Correlations were detected with Spearman’s correlation coefficient. All statistical analyses were performed with StatView or SAS statistical software (Cary, NC 27513, USA) and two-tailed p values below 0.05 were considered to denote significant differences.

Significance is noticed on Figures: * when p<0.05, * * when p<0.01 and * * * when p<0.001.

## Results

### Baseline CEC Counts

**Figure 1:**
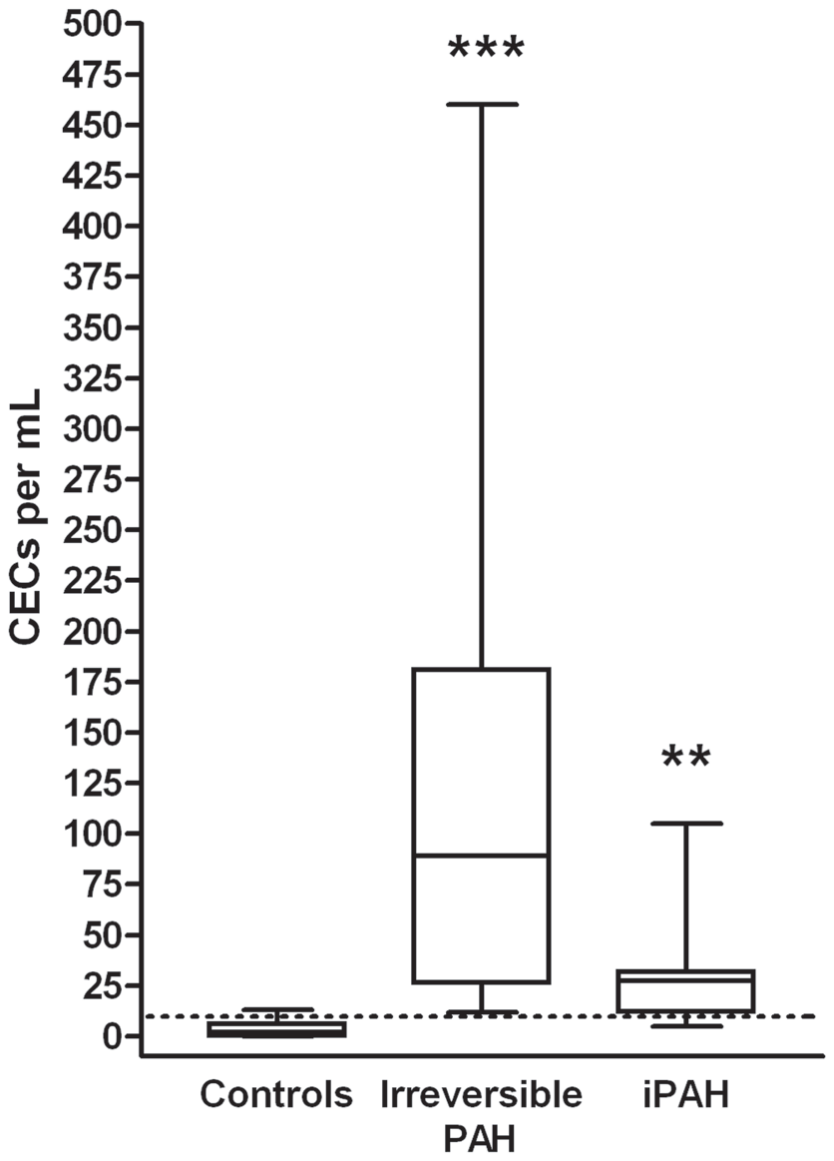
CECs are increased in irreversible and idiopathic pediatric PAH patients. CEC counts were significantly increased in irreversible and idiopathic PAH (iPAH). Effects of the group and their interaction on CEC variability were tested using ANOVA (***p = 0.0005 and **p = 0.01 for irreversible and idiopathic patients versus controls (reversible PAH), respectively.

Baseline was defined as the time when we decided to start PAH treatment in naïve incident patients or when we decided to add another drug in prevalent (already treated) patients. CEC counts at baseline were found higher in irreversible PAH-CHD (median 64 CECs per mL, range 12–242) compared to controls (median 2 CECs per mL, range 0–13, p = 0.0005, Figure 1). Furthermore, CEC level were also found higher compared to controls in pediatric idiopathic PAH (median 29 CECs per mL, range 0–460, p = 0.01 versus controls). CEC counts did not significantly differ between the two patient groups (p = 0.26 for PAH-HD versus iPAH). These results are in accordance with our previous results published in 2009 in a pediatric population and in 2010 in adult PAH [8], [10]. CEC counts did not correlate with age, O2 saturation, mean PAP or mean PVR. At baseline, 10 of the 15 patients with idiopathic PAH had increased level of CEC before treatment initiation as compared to reference range [12], while one patient had a normal level of CEC (5 CEC per mL) and the other one was in the upper range of normal values (9 CEC per mL). For irreversible PAH patients, all of them had increased CEC before oral treatment initiation. For patients receiving treprostinil, as shown in table 4, only 3 patients out of 10 had less than 10 CEC per mL of blood at baseline.

**Table 4:** CEC follow up in patients with SC treprostinil therapy.

Patient	CEC per mL at Baseline before adding SC- treprostinil	Treatment at baseline before adding SC- treprostinil	CEC per mL at H48 after adding SC-treprostinil	CEC per mL at Day 5 after adding SC- treprostinil	CEC per mL at 1 month after adding SC-treprostinil	Worsening after more than 1 month of SC-treprostinil 0 = no 1 = yes	CEC per mL at pre-worsening	CEC per mL at worsening (Months after pre-worsening)	clinical event at worsening	CEC per mL post worsening	action after worsening
1	6	**oral bitherapy**	5	0	0	0	/	/	/	/	/
2	79	**oral bitherapy**	0	0	2	1	0	79 (6 months)	NYHA IV	2	increasing doses of treprostinil
3	44	**oral bitherapy**	0	0	1	1	39	251 (6 months)	cyanosis, dyspnea	11	epoprostenol IV
4^†^	26	**oral bitherapy**	12	4	DC	/	/	/	/	/	/
5	32	**oral bitherapy**	6	6	10	1	15	122 (6 months)	NYHA IV	92	ductus arteriosus stenting and increasing doses of treprostinil
6^†^	0	**oral bitherapy**	0	0	13	1	13	164 (5 months)	DC	/	/
7	7	**epoprostenol IV**	0	15	4	0	/	/	/	/	/
8	13	**oral bitherapy**	2	/	/	/	2	32 (8 months)	dyspnea	1	increasing doses of treprostinil
9	43	**No treatment**	15	9	7	0	/	/	/	/	/
10	303	**oral bitherapy**	11	9	10	1	141	303 (1 month)	syncope	11	increasing doses of treprostinil

(^†^Deceased).

### CEC Counts in PAH Patients Treated with Oral Vasodilator Therapy

Fifty-two out of the 60 children receiving targeted PAH therapy received firstly oral monotherapy (bosentan n = 24, or sildenafil n = 26, and calcium channel blockers in 2 NO responders), 6 were treated with oral combined therapy (bosentan/sildenafil) and 2 with upfront tritherapy (bosentan/sildenafil/SC-treprostinil). During follow up of the 52 patients treated with oral monotherapy, 10 of them needed a second drug within a median delay of 18 months (range 0.5–36 months) and 8 patients deteriorated despite oral combined therapy and required prostacyclin analogue treatment (median delay of 36 months).

**Figure 2:**
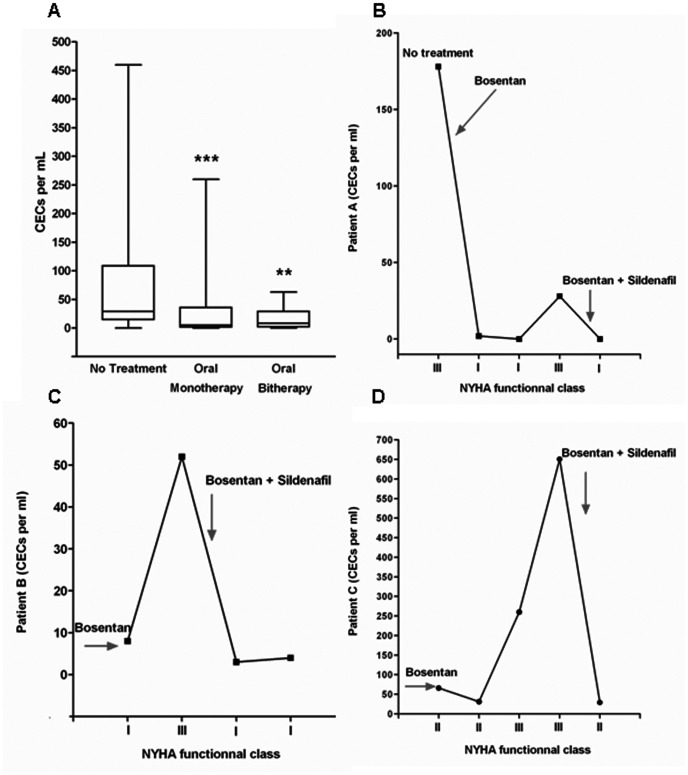
CEC counts in treated PAH. A- CEC counts were significantly reduced in treated PAH with oral mono- or bi-therapy in the absence of clinical worsening. Effects of group and their interaction on CEC variability were tested using ANOVA. Mono and combined oral therapy induced a significant decrease in CEC count as compared to patients in the absence of treatment (respectively ***p = 0.0007 and **p = 0.003). No difference was noticed between mono and combined therapy groups (p = 0.96 between mono and combined therapy). **B/C/D**- Time course of CEC count during PAH worsening in three patients treated with monotherapy (bosentan). Worsening was observed concomitantly to CEC level increase. Adding sildenafil to bosentan allowed a decrease in CEC levels to normal range values.

CEC levels were significantly reduced after treatment (mean decrease of 77%, p = 0.011 for all patients with oral mono or combined therapy *vs*. non treated patients) in all patients. When we analyzed clinically stable patients, we found that either oral mono or bitherapy significantly reduced CEC levels (Figure 2A, p = 0.0007 and 0.003 for mono and bitherapy respectively compared to patients without any treatment). In patients previously treated, CEC similarly decreased upon treatment. For patients under oral monotherapy, no significant difference was observed between idiopathic and irreversible PAH patients in terms of CEC number (irreversible PAH-CHD: median 7 CECs per mL, range 0–66; idiopathic PAH: median 32 CECs per mL, range 1–460; p = 0.27). Similarly, in patient receiving oral bitherapy, no significant difference in CEC number was observed between idiopathic or irreversible PAH patients (irreversible PAH-CHD: median 30 CECs per mL, range 0–34; idiopathic PAH: median 13 CECs per mL, range 0–166; p = 0.64). During follow-up, three Eisenmenger patients with monotherapy (bosentan) had clinical worsening. All these events appeared concomitantly to an increase in CEC counts. Adding sildenafil to bosentan drastically reduced the number of CECs, as shown in figure 2 B–D.

### CEC Counts in Worsening PAH Patients

Ten patients required prostacyclin analogues treatment, 2 of them upfront and 8 of them after worsening despite oral therapy. As previously described [7], the first treatment option in our center is currently subcutaneous (SC) treprostinil. Patient 7, in whom we switched from IV epoprostenol to subcutaneous treprostinil while she was in a stable condition, had low CEC counts while treated either with epoprostenol (baseline) or with treprostinil during the 3-year follow-up.

In the remaining 8 patients in whom we started treprostinil because of clinical worsening, CECs were counted at baseline before treprostinil and then at day 2, day 5, day 30 and every 3 months during follow-up. As shown in Table 4, all patients receiving treprostinil, whatever the initial treatment, had a decreased CEC number upon treatment initiation, and a further CEC increase was strictly correlated to modification of NYHA status. Only 3 patients out of 10 had less than 10 CEC per mL of blood at baseline. Treprostinil on top of combined oral treatment reduced CEC count within the first week in all patients (Table 4). All but one improved their clinical condition. This latter patient died of right heart failure one week after prostacyclin analogues had been initiated despite a decrease of CEC count from 26 to 4.

**Figure 3:**
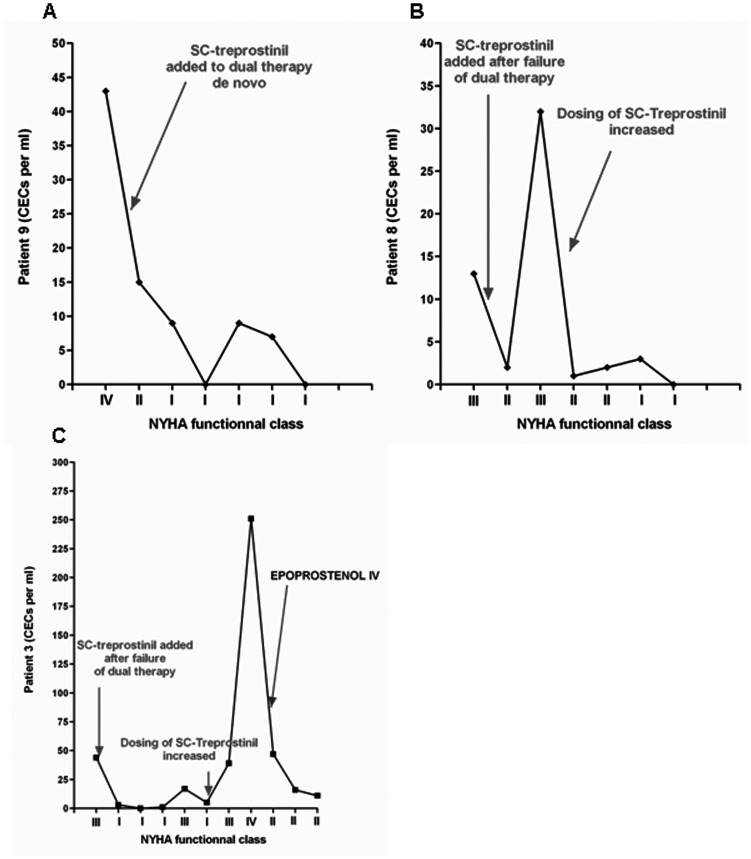
CEC modification during worsening in patients treated with SC treprostinil. A- Time course of CEC counts in a patient with stable iPAH under treatment. B- Time course of CEC counts in a patient with PAH-CHD with a small VSD (subtype 1C Dana-Point classification of CHD-PAH). C- Time course of CEC counts in a patient with iPAH.

During follow-up in the remaining 8 treprostinil-treated patients, another patient died after 6 months (patient 6). As shown in Table 4, CEC level decreased during the follow up to reach normal values. 3 of the 10 patients with treprostinil therapy (patient 1, 7 and 9) had stable low CEC levels while receiving treprostinil therapy, at least during the study period. To illustrate this observation, we have added the course of CEC level of patient 9 in Figure 3A. A severe CEC increase was observed (from 13 to 164/ml) before worsening and death. During follow-up of this combined therapy cohort, an increase in CEC counts was observed either before or at the time of clinical events, as shown in table 4. Examples of the CEC time course in an incident patient with PAH-CHD with a small VSD (subtype 1C Dana-Point classification of CHD-PAH) and in another patient with iPAH are shown respectively in figures 3B and 3C.

## Discussion

Endothelial cell dysfunction is a hallmark both of idiopathic PAH [13], [14], [15], [16] and of congenital heart disease related PAH [17]. Endothelial circulating biomarkers could thus be a novel non-invasive tool not only for diagnosis and/or prognosis but also to monitor treatment efficacy. Indeed, circulating endothelial cells (CECs) are recognized to be non-invasive markers of vascular damage, remodeling and dysfunction [12], [18], [19], [20]. Peripheral CEC counts are elevated in pulmonary hypertension of different etiologies [9], [10], including pulmonary hypertension secondary to congenital heart disease [8].

The present study therefore investigated the number of CECs after pulmonary vasodilator therapy in a pediatric PAH cohort. We show firstly that the number of CECs was markedly reduced by PAH-specific treatment in both patients with PAH-CHD or idiopathic PAH and, secondly, that an increase in CEC level during follow-up was associated with significant clinical deterioration. Importantly, the rises in CEC level was often observed prior clinical manifestations.

From a histopathological standpoint, “surgically irreversible” type PAH-CHD and idiopathic PAH are both associated with vascular remodeling, which includes endothelial and smooth muscle cell proliferation. Endothelial anomalies have a similar histological appearance in idiopathic and irreversible PAH-CHD [17], [21], [22]. CEC increase has been previously found in adult idiopathic PAH as well as in other forms of adult PH [9], [10]. We have shown that CECs were elevated in children with PAH-CHD and speculated previously that CEC count could be relevant in assessing reversibility of the pulmonary vascular disease after closure of the responsible left-to-right shunt [8]. Therefore, we hypothesized that CECs count could be used as a biomarker of clinical evolution in pediatric iPAH and in PAH-CHD.

In our study, the method used to quantify CECs considers the size and number of particles around the cells and allowed us to differentiate CECs from hematopoietic cells expressing CD146. Furthermore, Delorme et al. found that progenitor cells coexpressing CD146 and CD133, an immature hematopoietic antigen, looked different from mature CEC, the latter presenting morphologic features of differentiated cells and a size larger than 20 µm [11], [12], [23]. Therefore, we can speculate that CEC counted using the IMS method did not include progenitor cells. Moreover, in a previous work exploring effect of vasodilatator therapy on progenitor cells [24], we did not found any modification of hematopoietic derived progenitor cells CD133^+^CD34^+^ after treprostinil treatment whereas an increase of late endothelial progenitor cells (Late-EPCs or ECFC for endothelial colony forming cells [25], [26], [27]) colony numbers was observed. Here, inversely, we found a CEC decrease in the treprostinil groups, suggesting that ECFC numbers vary independently. Moreover, CEC numbers were found decreased after oral therapy, in contrast to ECFCs that were not influenced by oral vasodilatator therapy, but only after systemic prostacyclin analogues administration (subcutaneous injection of treprostinil). Finally, ECFC numbers did not allow us to discriminate treated and untreated patients (except for treprostinil which induced a cell increase) while CEC level allowed us to discriminate oral monotherapy, bitherapy and tritherapy with prostacyclin analogues. Mean values of ECFCs were from 0.3 per 5×10^6^ mononuclear cells in untreated PAH to 1.2 per 5×10^6^ mononuclear cells under treprostinil. Thus, a normal leukocyte count around 5×10^9^/L will give rise to ECFC numbers between 0.3 and 1.2 per mL, based on the hypothesis that 1 progenitor cell that gives rise to one colony, which, number is negligible compared to CEC counts (mean value of 73 CEC in non treated PAH).

In our series, CECs decreased in all patients after introduction of a new treatment, whatever the drug used either in monotherapy or in combination. This decrease in CEC count was observed both in incident patients who had never been treated with PAH drugs and in prevalent patients refered to our institution because of clinical worsening when already under PAH treatment. However, CECs remained above normal values in 2 patients with Eisenmenger syndrome, that could be related to a severe polycythemia and/or hyperviscosity in these subjects [28]. In the more severely affected children requiring addition of prostacyclin analogues, we also showed an early decrease in CEC count. The fact that subsequent increases in CECs was associated with worsening clinical status suggests that this may reflect accelerated endothelial remodeling/proliferation. Our results also suggest that treatement of PAH may slow or even stop this process.

It has been shown that CEC number correlates with other circulating markers of endothelial function [29]. These circulating factors, including ET-1, NO and VEGF have vasoactive effects but also play a crucial role in activation and proliferation of endothelial cells [30], [31], [32], [33]. PAH specific therapies are considered primarily as vasodilators because they block vasoconstriction (ERA) or promote vasodilation via cGMP (PDE5 inhibitors) or cAMP (prostacyclin analogues) pathways. They also play a role on vascular remodeling as shown in animal models and in vitro experiments [34], [35].

The unexpected rapid decrease of CEC counts after introduction of treprostinil could be mediated either by changes in vasoactive factor levels or by direct effects on damaged/activated endothelial cells. One of the other possibilities is the modulation of endothelial cell proliferation. Indeed, ET-1 is a vasoconstrictor but also a mitogen factor for endothelial cells [31], [32] and vascular smooth muscle cells [30], [33]. ERA such as prostacyclin analogues [33], [36] have been shown to decrease ET-1 production and proliferation of vascular cells into the lung. These treatments could also reduce inflammatory potential of endothelial cells [37] involved in endothelial remodeling.

One of our striking findings was that CEC count remains very low (normal or close to normal) in patients in whom our therapeutic goals could be reached (Figure 3A). Conversely, we have shown that patients who deteriorated during follow-up had acute increases in CEC counts that often preceded clinical worsening. In this study, we did not take CEC counts into consideration to modify therapy but it is noteworthy that increase in CEC counts occurred a few days or weeks before deterioration, in almost all worsening patients. This suggest that if CECs reflect endothelial damage/activation, their count may help to anticipate clinical deterioration. As shown in Figure 3B and C, CEC counts may vary during the natural course of PAH in these children and the periods of stability and worsening are clearly identified on the graph.

Clinical worsening remains the gold standard to treatment modification. However, a decreased level of CEC is effective when patients are treated and move from class II to class I where there is no BNP modification. For class III and IV, our results suggest that CEC level is modified at an early stage before BNP increase and could replace walking test in children who are not yet walking. In children with iPAH or PAH-CHD, the optimal timing of combination therapy is unclear. No data are readily available on “goal oriented treatment” in children. This could be explained by the heterogeneity of the population with regards to age, cause of PH and comorbidities such as Down syndrome in PAH-CHD. While applying a goal oriented treatment algorithm in children could potentially improve outcome, it is still challenging. Here, we show that CEC counts could be included in the panel of tests used to assess treatment responses in this population [2]. CEC counts should certainly not replace functional evaluation but could be a valuable adjunct to confirm a stable condition. Conversely, acute increase of CEC count in a previously stable patient should raise awareness of a potential future worsening and certainly prompt careful clinical assessment. Our data are not sufficient to claim that optimal PAH treatment should lead to normalization of CEC count. Hitherto, however, recent data on PAH treatment effect on mortality showed that improvement in survival is mainly driven by prevalent patients, but that improvement is less important in newly diagnosed incident pediatric patients. These findings suggest that optimization of therapy may consist of more aggressive treatment and potentially an earlier switch to combination therapy. To guide this optimization in incident iPAH pediatric patients, normal CEC counts could be used as a potential endpoint. Along the same line, patients in our series who had long term low CEC counts are those with the greatest clinical stability.

Here we show that CECs count can be a useful biomarker during follow-up of PAH treatment in pediatric iPAH and PAH-CHD. Larger cohorts of patients should be evaluated with this new tool to investigate whether could be used to optimize therapy in a goal oriented approach that include the whole panel of already available functional, structural and biomarker based tests.
