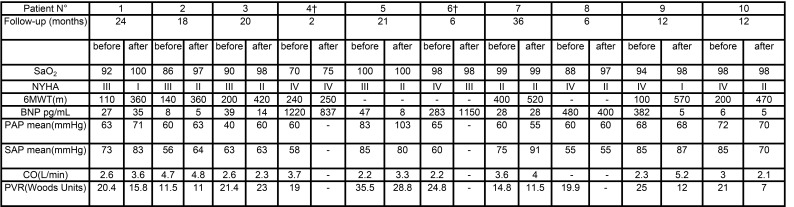# Correction: Circulating Endothelial Cells in Refractory Pulmonary Hypertension in Children: Markers of Treatment Efficacy and Clinical Worsening

**DOI:** 10.1371/annotation/ad9cc7fc-af50-4658-bd90-e3aaf0def017

**Published:** 2014-01-03

**Authors:** Marilyne Levy, Damien Bonnet, Laetitia Mauge, David S. Celermajer, Pascale Gaussem, David M. Smadja

An error was introduced in the preparation of this article for publication. Table 3 was only partially published. Please find a complete version of Table 3 here: 

**Figure:**